# Assessing changes to the fecal microbiota in dogs undergoing elective orthopedic surgery: A preliminary investigation

**DOI:** 10.1371/journal.pone.0325163

**Published:** 2025-06-02

**Authors:** Allison J. Collier, Diego E. Gomez, Melissa A. MacIver, Adronie Verbrugghe, J. Scott Weese, Shauna L. Blois

**Affiliations:** 1 Department of Clinical Studies, Ontario Veterinary College, University of Guelph, Guelph, Ontario, Canada; 2 Abu Dhabi Equine and Camel Hospital, Al Wathba, Abu Dhabi, United Arab Emirates; 3 Centre for Public Health and Zoonoses, Ontario Veterinary College, University of Guelph, Guelph, Ontario, Canada; University of Illinois, UNITED STATES OF AMERICA

## Abstract

**Background:**

Studies assessing the impact of surgery on the canine gut microbiota are limited. This study assessed the fecal microbiota before and up to 3 months after elective orthopedic surgery.

**Methods:**

Fourteen client-owned dogs >1 year of age undergoing elective orthopedic surgery were recruited. Dogs received perioperative antibiotics only (perioperative cefazolin, n = 7) or were discharged with oral cephalexin following surgery for 5–7 days (n = 7) in conjunction with perioperative antibiotics. Fecal samples were collected at baseline and at recheck 1 (13–50 days post-operatively) and recheck 2 (55–90 days post-operatively). The fecal microbiota was analyzed using 16S amplicon sequencing. Alpha diversity was assessed with the Sobs Index, Shannon Diversity Index, and Inverse Simpson Index, whereas beta diversity was assessed with the Bray-Curtis Index and Jaccard Index.

**Results:**

In the perioperative and post-operative antibiotic groups, the Inverse Simpson and Shannon Diversity Index differed between baseline and recheck 1 (p < 0.05), baseline and recheck 2 (p < 0.05), but not between recheck 1 and recheck 2 (p > 0.05). The Sobs Index was only significantly different between baseline and recheck 1 (p = 0.02) in both groups. The Bray-Curtis and Jaccard Index were significantly different at rechecks 1 and 2 compared to baseline (p > 0.05) in the post-operative antibiotic group but not in dogs that received only perioperative antibiotics. Both the Bray Curtis and Jaccard Index were significantly different between the antibiotic prescription types (p = 0.001) although measures of alpha diversity were not (p > 0.05).

**Conclusions:**

Alterations in community structure, richness, and diversity were identified in dogs undergoing elective orthopedic surgery, with many changes persisting at least 2–3 months post-operatively in dogs receiving perioperative and/or post-operative antibiotics.

## Introduction

The gastrointestinal (GI) microbiota consists of a highly diverse array of bacteria, fungi, protozoa, viruses, and other essential factors within the GI tract [1–3]. The GI microbiota has several important functions in both health and disease, as it plays an essential role in processes such as promoting enterocyte turnover, mucous production, short-chain fatty acid production, defense against enteropathogens, and promoting self-tolerance, amongst other essential factors [4,5]

Dysbiosis and alterations to the GI microbiota can occur in many disease states, including obesity, inflammatory bowel disease, inflammation, and immune mediated disease, amongst other causes [2,6–11]. Dysbiosis can also occur subsequent to many medications, including antibiotics and antacids, in particular [12–17]. Surgical patients are at risk for dysbiosis for numerous reasons, including changes in diet and fasting requirements, changes in activity levels, the potential use of perioperative antibiotics, stress, inflammation, and ileus, along with other contributing factors [18–21]. Alterations to the GI microbiota have been noted in people following surgical procedures and could be associated with post-operative complications [20,22–24].

The microbiota has also been shown to play an important role in orthopedic disease in people, including bone growth, regulation of bone homeostasis, and impacting the progression of osteoarthritis [25–27]. Alterations to the GI microbiota have been seen in people following orthopedic surgery [28]. The impact of orthopedic surgery on the canine GI microbiota is unknown, and investigations of the impact of surgery on the GI microbiota in companion animals are lacking. Post-operative alterations to the GI microbiota are suspected in dogs undergoing surgery, particularly given the high rate of perioperative or postoperative antibiotic use in these patients [29].

The objective of this study was to evaluate the fecal microbiota in dogs before and after elective orthopedic surgery and to evaluate the impact of antimicrobial prophylaxis regimen on the fecal microbiota.

## Materials and methods

### Study population

Dogs 1 year of age or older admitted to the Ontario Veterinary College Health Sciences Centre (OVC-HSC) for any elective orthopedic surgery between February 2022 and February 2023 were considered eligible for inclusion in this prospective cohort study. All dogs were privately owned, the owners signed an informed consent, and the study was approved by the University of Guelph Animal Care Committee (AUP #4839). Exclusion criteria included reported vomiting and diarrhea within 1 month of admission, clients who elected not to return to OVC for recheck examinations, a history of chronic enteropathy/inflammatory bowel disease, a history of consuming raw meat, or administration of any antibiotic, probiotic, prebiotic, synbiotic, and/or immunosuppressive medication within 1 month before admission.

Fresh fecal samples (or rectal swabs if a fresh fecal sample was not available) were collected from all dogs on admission or within 24 hours before the elective orthopedic surgery. A second fresh fecal sample or rectal swab was obtained at the first recheck, approximately 1 month following surgery, and a third sample was obtained at the second recheck examination, approximately 2–3 months following surgery. All medication and supplement use, including antibiotics and probiotics, was recorded at each visit. Dogs were also grouped based on whether they had received perioperative antibiotics only (cefazolin 22 mg/kg intravenously every 90 minutes from initiation (30–60 minutes prior to the procedure) until the end of the procedure) (perioperative group) or received post-operative antibiotics (cephalexin 22 mg/kg per os (PO) every 12 hours) in addition to perioperative antibiotics (post-operative group). Dogs were considered to have received a post-operative course of antibiotics if they were prescribed oral antibiotics to go home, in addition to receiving perioperative antibiotics. Dogs were considered to have received perioperative antibiotics if they only received injectable antibiotics shortly (30–60 minutes) before the procedure, stopped within 24 hours of the end of the procedure, and did not receive any post-operative antibiotics.

When handling fecal samples or collecting rectal swabs, gloves were worn. The rectal swab sample was obtained by inserting a sterile cotton-tipped swab approximately 2–3 cm into the anus. The swab was rotated 360 degrees, then removed. Fecal material was visually confirmed to be present on the swab before it was placed in a sterile tube. The tube or fresh fecal sample was immediately stored at -20C until further analysis.

### Microbiota analysis

Prior to deoxyribonucleic acid (DNA) extraction, fecal samples were brought to room temperature for approximately 1–2 hours. DNA extraction was performed using the E.Z.N.A. Stool DNA Kit Pathogen Detection Protocol (Omega Bio-Tek Inc., Doraville, Georgia, USA) performed according to the manufacturer’s instructions. Following DNA extraction, samples were stored at -20C until polymerase chain reaction (PCR). The DNA samples were submitted to the Agriculture and Food Laboratory, Laboratory Services Division (University of Guelph, Guelph, Ontario, Canada) for amplicon library preparation and sequencing. The libraries were prepared based on amplification of the V4 region of the 16S rRNA gene through PCR. The locus specific primers included the following: forward primer: 5’-GTG YCA GCM GCC GCG GTA A-3’, and reverse primer: 5’-GGA CTA CNV GGG TWT CTA AT-3’. The libraries were then sequenced using an Illumina MiSeq platform with a MiSeq Reagent Kit v2 and 2x250 paired-end cycles according to the manufacturer’s protocol (Illumina, San Diego, California, USA). Analysis of sequencing data was carried out using the software Mothur v1.48.0 using a previously described and established protocol [30]. In summary, paired-end reads were assembled with the make.contigs command. Sequences greater than 250 base pairs were removed, along with sequences containing ambiguous base pairs or with homopolymers longer than 8 bp. Sequences were aligned against the SILVA reference database. Chimeras were removed [31]. The Ribosomal Database Project classifier (v14) was used for taxonomic assignment of sequences. Sequences were also classified into operational taxonomic units (OTUs).

### Statistical analysis

The Shapiro Wilk Test in RStudio (v2023.12.1) [32] was utilized to assess for normality. Any data that did not demonstrate a normal distribution were log transformed to meet the assumptions of normality. Alpha diversity was assessed using richness (Sobs Index calculation in Mothur (v1.48.0) demonstrating the number of observed species), and the Inverse Simpson and Shannon Diversity indices. Beta diversity was assessed using the Bray-Curtis and the Jaccard indices in Mothur. Statistically significant differences in beta diversity were assessed through the analysis of molecular variance (ANOVA) test in Mothur and compared among groups by Permutational Multivariate Analysis of Variance (PERMANOVA) using the adonis2 function in RStudio (v2023.12.1). Significant differences in alpha diversity were assessed through an ANOVA and Tukey’s Honest Significant Difference test in RStudio (v2023.12.1) [32]. Factorial ANOVA 2 (with the ez package) in RStudio was utilized to assess the impact of the interaction of time and antibiotic prescription type (perioperative only vs post-operative antibiotic therapy) on alpha diversity metrics. To assess for differences in the relative abundances of different phyla, families, and genera, the Kruskal-Wallis rank sum test was utilized with Benjamini and Hochberg adjustment of p-values for multiple comparisons. Significance was set at p or q < 0.05 for all analyses.

## Results

### Study population characteristics

Fourteen dogs were enrolled. The median age at presentation was 4.2 years (range 1.0–7.1 years). Breeds included a Maltese (n = 1), Toy Poodle (n = 1), Irish Setter (n = 1), German Shepherd (n = 1), Pomeranian (n = 1), Leonberger (n = 1), Polish Sheepdog (n = 1), and mixed breed (n = 7). The orthopedic procedure being performed included medial patellar luxation (MPL) repair (n = 3), osteochondrosis dessicans (OCD) surgery (n = 1), tibial plateau levelling osteotomy (TPLO) (n = 7), extra-capsular repair (n = 1), femoral head ostectomy (FHO) (n = 1), and bilateral elbow arthroscopy (n = 1). A fresh fecal sample or rectal swab was obtained on all dogs at baseline, recheck 1 (13–50 days post-operatively), and recheck 2 (55–90 days post-operatively). Characteristics of the enrolled dogs are further illustrated in Table 1.

**Table 1:** Characteristics of dogs (n = 14) undergoing orthopedic surgery and enrolled in a study investigating the fecal microbiota 1 month (recheck 1) and 2-3 months (recheck 2) post-surgery.

Dog	Procedure	Breed	Age (years)	Sex	Recheck 1 date (days)	Recheck 2 date (days)	Antibiotics received
1	MPL	Maltese	1.3	MN	37	79	Perioperative
2	OCD	Mixed	1.0	FS	41	76	Perioperative
3	TPLO	Mixed	6.8	MN	34	83	Post-operative
4	MPL	Mixed	1.3	FS	50	85	Perioperative
5	Extra-capsular repair	Toy poodle	3.6	FS	43	90	Perioperative
6	TPLO	Mixed	7.7	FS	28	55	Post-operative
7	TPLO	Irish setter	4.8	MN	36	76	Post-operative
8	TPLO	German shepherd	5.0	MN	34	90	Post-operative
9	TPLO	Mixed	8.1	FS	28	70	Post-operative
10	MPL	Pomeranian	1.3	FS	35	62	Perioperative
11	FHO	Mixed	1.1	FS	14	55	Perioperative
12	TPLO	Leonberger	6.7	FS	44	84	Post-operative
13	TPLO	Polish sheepdog	7.6	MN	15	60	Post-operative
14	Arthroscopy	Mixed	1.0	MN	13	60	Perioperative

Age is shown in years, and recheck dates are shown in days post-surgery. Patients were also grouped as to whether they received perioperative antibiotics only (perioperative cefazolin), or whether they also received post-operative antibiotics (Cephalexin for 5–7 days) in addition to perioperative antibiotics. Sex is shown as female spayed (FS) or male neutered (MN).

All 14 of the dogs had received antibiotics either in the perioperative period only (n = 7) or both peri- and post-operatively (n = 7). All dogs in the perioperative group received cefazolin (22 mg/kg) intravenously every 90 minutes from initiation (30–60 minutes prior to the procedure) until the end of the procedure. All dogs in the post-operative group received perioperative cefazolin (22 mg/kg) followed by a 5–7 day course of oral cephalexin (doses 22.0–27.8 mg/kg PO BID)(n = 7).

Complications were noted throughout the study period in four dogs. Complications included stifle instability following extra-capsular repair of cranial cruciate ligament injury (n = 1), tibial tuberosity fracture following TPLO (n = 1), lateral patellar luxation following MPL repair (n = 1), and fibular fracture following TPLO (n = 1). None were treated with antimicrobials for these complications. No surgical site infections were noted throughout the study period.

### Microbiota results

A total of 2,653,068 good quality reads were used for final analysis (median 64,808/sample, range 5,646−117,161/sample). A subsample of 5,646 reads was used based on the sample with the smallest number of reads. The median Good’s Coverage Index was 99.34% (98.68%−99.61%).

#### Alpha diversity.

**Fig 1:**
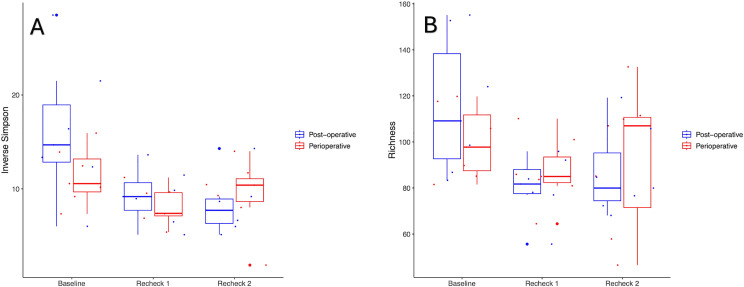
Inverse Simpson index (A) and richness (B) shown for the post-operative and perioperative groups. Significant decreases in the Inverse Simpson index (A) were noticed between Baseline and Recheck 1 (p = 0.01 perioperative group, p = 0.04 post-operative group), as well as between Baseline and Recheck 2 (p = 0.01 perioperative, p = 0.02 post-operative) in both the perioperative and post-operative groups. Significant differences were noted in richness (B) overall between time points (p < 0.05) in both the perioperative and post-operative group, and specifically between Baseline and Recheck 1 (p = 0.02 in perioperative and p = 0.01 in the post-operative group), but not between the other time points.

Significant differences were seen in alpha diversity over time in both groups. Richness (Sobs Index) decreased at recheck 1 compared to baseline in the perioperative and post-operative groups (p = 0.02 p = 0.01, respectively), but not between baseline and recheck 2 (p = 0.09 and p = 0.06 respectively) and recheck 1 and recheck 2 (p = 0.76 and p = 0.84 respectively). The Inverse Simpson Index significantly decreased at rechecks 1 and 2 compared to baseline in both groups (p = 0.01 and p = 0.01 for the perioperative group, p = 0.04 and p = 0.02 for the post-operative group) but not between recheck 1 and recheck 2 (p = 0.99 for the perioperative group and p = 0.86 for the post-operative group). The Shannon Diversity Index decreased at rechecks 1 and 2 compared to baseline in both the perioperative (p = 0.02 and p = 0.03) and post-operative (p = 0.02 and p = 0.01) groups, but not between recheck 1 and recheck 2 (p = 0.90 in the perioperative and p = 0.97 in the post-operative group). Changes in alpha diversity are further illustrated in Fig 1.

The comparison of the alpha diversity indices between dogs that had received perioperative only as compared to post-operative antibiotics did not show significant differences in richness (Sobs Index) (p = 0.89), the Shannon Diversity Index (p = 0.38), or the Inverse Simpson Index (p = 0.43). Additionally, the interaction between time and antibiotic prescription type (perioperative only vs post-operative antibiotic therapy) did not significantly influence richness (Sobs Index) (p = 0.30), the Shannon Diversity Index (p = 0.28), or the Inverse Simpson Index (p = 0.39).

#### Beta diversity.

**Fig 2:**
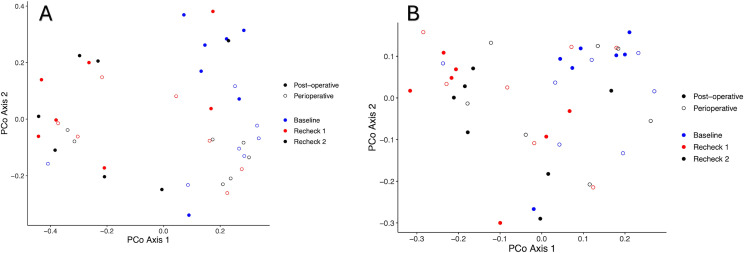
PCoA of the Bray-curtis (A) and Jaccard Index (B) in the perioperative and post-operative groups. Significant differences were noted between baseline and recheck 1 (p = 0.01) as well as baseline and recheck 2 (p = 0.01), but not between recheck 1 and recheck 2 (p > 0.05) for the Bray-Curtis Index (A) in dogs that received post-operative antibiotics. Significant differences were noted between baseline and recheck 1 (p = 0.002) as well as baseline and recheck 2 (p = 0.01), but not between recheck 1 and recheck 2 (p > 0.05) for the Jaccard Index (B) in dogs that received post-operative antibiotics. No significant differences were noted for either index in dogs that received perioperative antibiotics (p > 0.05).

When comparing beta diversity across the different time points, the Bray-Curtis and Jaccard Indices significantly differed overall for the post-operative antibiotic group. In-particular, they were significantly different between baseline and recheck 1 (p = 0.01 and p = 0.002, respectively), baseline and recheck 2 (p = 0.01 and p = 0.01, respectively), but not between recheck 1 and recheck 2 (p = 0.97 and p = 0.84, respectively). The Jaccard Index and Bray-Curtis Index did not differ between the time points in dogs that received perioperative antibiotics (p > 0.05). This is further illustrated in Fig 2.

The comparison of dogs that received perioperative and post-operative antibiotics showed significant differences in the Bray-Curtis Index (p = 0.001) and Jaccard Index (p = 0.001) between groups. The interaction between time and antibiotic prescription type (perioperative only vs post-operative antibiotic therapy) did not significantly influence the Bray-Curtis Index (p = 0.12).

#### Relative abundance.

**Fig 3:**
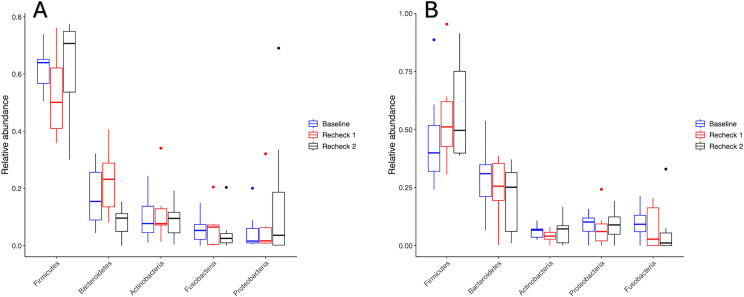
Relative abundance of the 5 most prevalent phyla in perioperative (A) and post-operative (B) groups. There were no significant differences in either group in the relative abundance of phyla between the different time points (q > 0.05).

**Fig 4:**
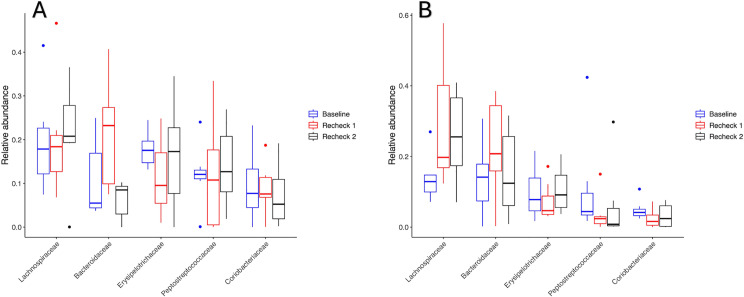
Relative abundance of the 5 most prevalent families in perioperative (A) and post-operative (B) groups. There were no significant differences in either group in the relative abundance of these families between the different time points (q > 0.05).

**Fig 5:**
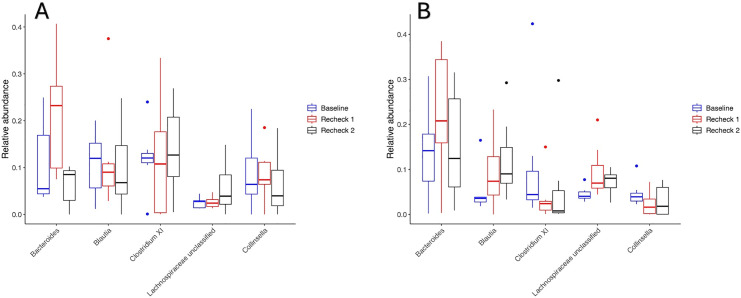
Relative abundance of the 5 most prevalent genera in perioperative (A) and post-operative (B) groups. There were no significant differences in the relative abundance of genera between the different time points in either group (q > 0.05).

**Fig 6:**
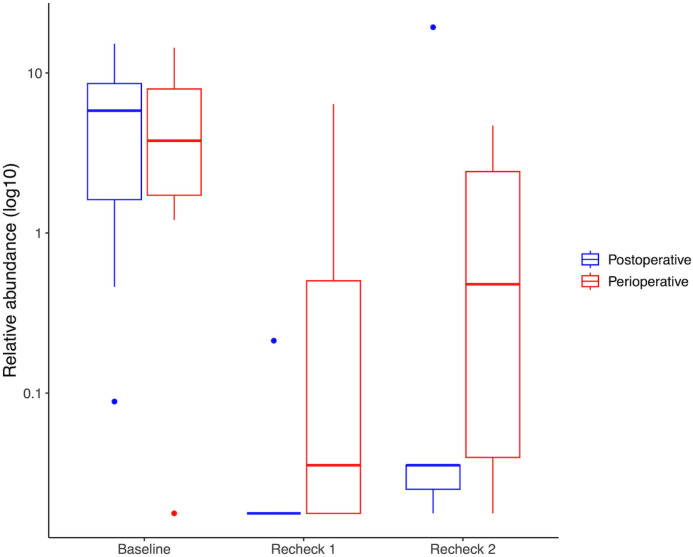
Relative abundance of Prevotellaceae in the perioperative and post-operative groups. The relative abundance of Prevotellaceae is significantly different between the different time points in the post-operative group (q=0.049), with the highest relative abundance at baseline. There were no significant differences in the relative abundance of Prevotellaceae in the perioperative group.

The relative abundances of phyla, families, and genera were assessed, and the top 5 most abundant taxa are demonstrated in Figs 3–5 respectively. No differences occurred in the relative abundance at the phylum level (q-value > 0.05, for all comparisons in the perioperative and post-operative group) or at the genus level (q-value > 0.05, for all comparisons in the perioperative and post-operative group) between the time points. However, the relative abundance of the family Prevotellaceae differed among time points in the post-operative group (Fig 6) (q-value = 0.049), having a higher relative abundance at baseline than recheck 1 and recheck 2. The relative abundance of family Propionibacteriaceae and Helicobacteraceae were significantly higher in dogs that received perioperative antibiotics only than those that received post-operative antibiotics (q-value of 0.002 and 0.04 respectively).

## Discussion

This study describes the fecal microbiota following elective orthopedic surgery in dogs that received perioperative with or without post-operative cephalosporin antibiotics. Significant alterations to the GI microbiota (in terms of community composition, structure, richness, and diversity) occurred in dogs undergoing elective orthopedic surgery, with many changes persisting at the time of the second recheck examination, 2–3 months post-operatively.

Dogs receiving post-operative antibiotics displayed significant changes in beta diversity over time, although a similar change was not observed in the dogs receiving only perioperative antibiotics. The change in beta diversity of dogs receiving post-operative antibiotics persisted for at least 2–3 months following surgery. This contrasts with the decrease in measures of alpha diversity (Inverse Simpson and Shannon Diversity indices) observed in all dogs regardless of the duration of antibiotic therapy given. This could suggest that perioperative and post-operative antibiotics have a similar impact on alpha diversity (including richness and evenness), but that post-operative antibiotics have a more significant impact on community membership and structure. However, given the small sample size, further analysis is needed in this area.

Dogs undergoing elective orthopedic surgery have many factors and interventions that could contribute to the observed alterations in the GI microbiota. Antibiotic use is one of the main factors likely influencing the fecal microbiota and leading to decreases in richness and diversity in this population of dogs undergoing elective orthopedic surgery. The detrimental impact of antibiotics on the GI microbiota and the resulting decreases in richness and diversity have been shown in numerous studies on humans, horses, and dogs [12–14,33–39]. In healthy dogs, administration of metronidazole at 12.5 mg/kg twice daily results in significant alterations to the GI microbiota, including a reduction in bacterial diversity [14,34]. However, differences between studies regarding the recovery of the microbiota are reported, with a study showing recovery of alpha diversity indices to baseline levels 14 and 28 days after cessation of antibiotics [14]. In contrast, another study reported that changes did not uniformly resolve even 4 weeks after administration, although longer time points were not assessed [34]. Additionally, administering 20 mg/kg of tylosin (macrolide class) for one week to healthy dogs has been shown to decrease bacterial diversity and richness on day 7, however by day 63, bacterial taxa were not significantly different compared to baseline, although the extent of microbial recovery was individualized [35]. Our study showed that the changes in the fecal microbiota following cephalosporin administration and orthopedic surgery can have persistent and significant alterations 55–90 days following elective orthopedic surgery where perioperative or post-operative antibiotics are utilized. The underlying reasons for the differences between studies regarding the duration of the microbial changes remain unclear, but other factors, such as fasting, post-operative inflammation, stress, or changes in activity level, may have influenced the fecal microbiota in these dogs compared to antibiotic use alone [5,21,40].

Changes in activity level could be another factor impacting the GI microbiota in dogs undergoing orthopedic surgery. Owners are typically advised to restrict their dog’s exercise for approximately 8–12 weeks following orthopedic surgery. Alterations to the GI microbiota through enhancement of richness and diversity have been shown in people with physical activity [41]. The impact of changes in physical activity on the canine GI microbiota remains to be determined. However, it is possible this was a factor resulting in some alterations to the GI microbiota in this population of dogs undergoing elective orthopedic surgery.

Changes in the GI microbiota (in terms of community composition and structure) also occurred between patients that received perioperative only versus those that also received post-operative antibiotics. In our study, Propionibacteriaceae and Helicobacteriaceae were significantly more abundant in patients that had received perioperative antibiotics alone compared to those receiving post-operative oral cephalexin. Metronidazole treatment in healthy dogs reduced the relative abundance of several families—including Bacteroidaceae, Clostridiaceae, Fusobacteriaceae, Lachnospiraceae, Ruminococcaceae, Turicibacteraceae, and Veillonellaceae—while increasing levels of Bifidobacteriaceae, Enterobacteriaceae, Enterococcaceae, and Streptococcaceae, all of which returned to baseline proportions by day 42 [14]. Similarly, amoxicillin administration in healthy dogs resulted in a decrease of taxa from the families Succinivibrionaceae and Bacteroidaceae, alongside an increase in Enterobacteriaceae [42]. Additionally, Helicobacteriaceae has been shown to decrease with antibiotic treatment in other species and potentially were more susceptible to the more prolonged post-operative antibiotic treatment courses compared to perioperative antibiotic treatment only [43]. These findings suggest that different classes of antibiotics exert distinct effects on various taxa, although there are no known studies comparing the impact on the fecal microbiota of perioperative only as compared to post-operative antibiotics. The findings from our study could indicate a differing impact on the GI microbiota in dogs that receive perioperative antibiotics only as compared to post-operative antibiotics. This could be of interest, as alterations to the GI microbiota could potentially alter the risk of surgical site infections, or alter the risk of antibiotic associated adverse events [44–47]. However, the clinical implications of the persisting alterations to the GI microbiota in dogs, and how it may alter the incidence of adverse events or surgical site infections, are unknown and further investigation is warranted in this area.

Several limitations were present in the current study. Convenience sampling was used, leading to limited assessments of the GI microbiota at time points that the dogs were coming to the hospital for the surgery or planned rechecks at approximately 1 month and 2–3 months following surgery. Therefore, the longer-term impact of surgery and antibiotics on the GI microbiota could not be assessed, nor were time points within the first weeks following surgery. Another limitation of the study was the small sample size, which limited the statistical power. However, this study can serve as a preliminary investigation for which to build future research on potential factors impacting the fecal microbiota in dogs undergoing elective orthopedic surgery, as well as to assess differences in the impact of perioperative compared to postoperative antibiotic therapy. Other limitations included the fact the dogs were a heterogenous population of client-owned dogs with different lifestyles and fed varying diets. As diet is one of the chief factors influencing the GI microbiota [5], this likely had an impact on the fecal microbiota in this study. However, the dogs were kept on a consistent diet throughout the study period, limiting intra-individual variations related to diet. As well, changes in body condition were not recorded throughout the study and could have impacted fecal microbiota results. Additionally, all dogs received antibiotics in this study, and therefore the impact of orthopedic surgery alone on the GI microbiota could not be determined. However, this limitation is difficult to avoid, as antibiotics are routinely administered to patients undergoing orthopedic procedures to reduce the risk of surgical site infections [29].

In conclusion, decreases in richness and diversity were seen in the fecal microbiota of dogs undergoing elective orthopedic surgery and receiving either post-operative or only perioperative cephalosporin class of antibiotics, with many changes persisting at the time of the second recheck examination, 2–3 months post-operatively. Therefore, this study highlights that a sustained impact can be seen in the GI in dogs undergoing elective orthopedic surgery receiving perioperative and post-operative antibiotics, although the clinical implications of this effect on the fecal microbiota are currently unknown. Further investigation is needed to explore reasons for the persisting changes seen in the GI microbiota following elective orthopedic surgery, and to better determine if there are differences to the GI microbiota in patients that receive post-operative antibiotics as compared to perioperative antibiotics only.

## Supporting information

S1 Table Richness (Sobs Index), Inverse Simpson, and Shannon Indices for the perioperative and post-operative groups. (DOCX)
