# Supplementary material for: Assessing changes to the fecal microbiota in dogs undergoing elective orthopedic surgery: A preliminary investigation
Source: PLoS One. 2025 Jun 2;20(6):e0325163. doi: 10.1371/journal.pone.0325163 (PMC12129194; doi:10.1371/journal.pone.0325163)
Supplement: S1 Table — (DOCX) [file pone.0325163.s001.docx]

| **Alpha diversity metric** | **Group** | **Comparison** | **p-value** |
| --- | --- | --- | --- |
| Richness (Sobs Index) | Perioperative | Baseline- Recheck 1 | 0.02 |
| Richness (Sobs Index) | Post-operative | Baseline-Recheck 1 | 0.01 |
| Richness (Sobs Index) | Perioperative | Baseline-Recheck 2 | 0.09 |
| Richness (Sobs Index) | Post-operative | Baseline-Recheck 2 | 0.06 |
| Richness (Sobs Index) | Perioperative | Recheck 1- Recheck 2 | 0.76 |
| Richness (Sobs Index) | Post-operative | Recheck 1-Recheck 2 | 0.84 |
| Inverse Simpson | Perioperative | Baseline- Recheck 1 | 0.01 |
| Inverse Simpson | Post-operative | Baseline-Recheck 1 | 0.04 |
| Inverse Simpson | Perioperative | Baseline-Recheck 2 | 0.01 |
| Inverse Simpson | Post-operative | Baseline-Recheck 2 | 0.02 |
| Inverse Simpson | Perioperative | Recheck 1- Recheck 2 | 0.99 |
| Inverse Simpson | Post-operative | Recheck 1-Recheck 2 | 0.86 |
| Shannon | Perioperative | Baseline- Recheck 1 | 0.01 |
| Shannon | Post-operative | Baseline-Recheck 1 | 0.02 |
| Shannon | Perioperative | Baseline-Recheck 2 | 0.03 |
| Shannon | Post-operative | Baseline-Recheck 2 | 0.02 |
| Shannon | Perioperative | Recheck 1- Recheck 2 | 0.90 |
| Shannon | Post-operative | Recheck 1-Recheck 2 | 0.97 |
